# Plant Species and Functional Group Combinations Affect Green Roof Ecosystem Functions

**DOI:** 10.1371/journal.pone.0009677

**Published:** 2010-03-12

**Authors:** Jeremy Lundholm, J. Scott MacIvor, Zachary MacDougall, Melissa Ranalli

**Affiliations:** Biology Department, Saint Mary's University, Halifax, Nova Scotia, Canada; University Copenhagen, Denmark

## Abstract

**Background:**

Green roofs perform ecosystem services such as summer roof temperature reduction and stormwater capture that directly contribute to lower building energy use and potential economic savings. These services are in turn related to ecosystem functions performed by the vegetation layer such as radiation reflection and transpiration, but little work has examined the role of plant species composition and diversity in improving these functions.

**Methodology/Principal Findings:**

We used a replicated modular extensive (shallow growing- medium) green roof system planted with monocultures or mixtures containing one, three or five life-forms, to quantify two ecosystem services: summer roof cooling and water capture. We also measured the related ecosystem properties/processes of albedo, evapotranspiration, and the mean and temporal variability of aboveground biomass over four months. Mixtures containing three or five life-form groups, simultaneously optimized several green roof ecosystem functions, outperforming monocultures and single life-form groups, but there was much variation in performance depending on which life-forms were present in the three life-form mixtures. Some mixtures outperformed the best monocultures for water capture, evapotranspiration, and an index combining both water capture and temperature reductions. Combinations of tall forbs, grasses and succulents simultaneously optimized a range of ecosystem performance measures, thus the main benefit of including all three groups was not to maximize any single process but to perform a variety of functions well.

**Conclusions/Significance:**

Ecosystem services from green roofs can be improved by planting certain life-form groups in combination, directly contributing to climate change mitigation and adaptation strategies. The strong performance by certain mixtures of life-forms, especially tall forbs, grasses and succulents, warrants further investigation into niche complementarity or facilitation as mechanisms governing biodiversity-ecosystem functioning relationships in green roof ecosystems.

## Introduction

Ecological engineers and landscape architects have begun to construct ecosystems that provide a range of services, including wastewater treatment [1], [2], removal of contaminants from indoor air [3], stormwater retention [4] and provision of habitat for native biodiversity [5]. Many studies in natural [6], [7] and agricultural [8] ecosystems have shown that higher levels of taxonomic and functional diversity in biological communities result in greater provisioning of ecosystem services, as well as higher rates of resource uptake and other processes. In contrast, very little work has been done to evaluate the potential role of biodiversity in the ecosystem functioning of engineered or constructed ecosystems such as sewage treatment wetlands, biofilters and green roofs [9]–[11].

Built environments account for at least 50% of the total energy consumed by human societies [12] and they have direct impacts on regional climates through the urban heat island effect [13]. Commercial and residential buildings are responsible for approximately 7.9% of greenhouse gas emissions and the heating and cooling of buildings consumes up to 20% of the total energy used in developed countries [14]. Recognizing these substantial impacts, the building industry now prioritizes energy savings at the building level to reduce the carbon footprint and the overall environmental impact of cities [15].

Impermeable concrete and asphalt surfaces in urban areas also exacerbate stormwater runoff and increase erosion [16], [17]. Among hard building surfaces, roofs account for up to 60% of building cooling load [18], and can represent up to 30% of urban impervious surfaces [19], thus roofs make substantial contributions to both energy consumption and stormwater runoff problems. Green roofs replace some of the functions lost when natural areas are transformed into buildings, by converting unused space on rooftops into vegetated ecosystems. Green or living roofs consist of a growing medium and vegetation layer, over engineered roof membranes [20]. While green roofs have a long history [21], their construction has increased substantially in recent years. Despite the recent economic downturn, the number of green roof installations in North America increased 35% in 2008 [22]. While these constructed ecosystems contribute many services to the urban environment, the reduction of heat flux into buildings during hot seasons [23]–[25] and the capture and retention of stormwater are the best known and most investigated [17], [26], [27]. Green roofs are also expected to reduce the urban heat island effect, if deployed on a large scale [28], [29].

The vegetation layer contributes to roof cooling by reflecting and absorbing solar radiation [30], and through evapotranspiration [25], but the effects of different vegetation composition on green roof performance have received little empirical evaluation [20]. Greenhouse studies have suggested that plant species composition can affect green roof functions such as stormwater capture [31] and water loss through evapotranspiration [32], but this has rarely been examined on actual roofs and no studies examining the effects of plant taxonomic or functional diversity on green roof services have been published. Evapotranspiration and albedo may be affected by ecosystem properties such as aboveground biomass or leaf area index [33], as well as morphological and physiological differences between plant species [34], [35]. Ecosystem properties may in turn be affected by community properties such as the diversity of plant species, which has been shown to increase productivity in a number of terrestrial ecosystems [36], [37]. Additionally, since many green roofs have very shallow growing medium layers, the temporal stability of vegetation cover in such a drought-prone habitat is itself an important service that may also be promoted by greater species diversity.

Plant life-forms such as grasses, shrubs and forbs represent different life history strategies, resource use patterns, and suites of adaptations to the external environment [38]. Plant life-form diversity can be considered a coarse surrogate for the functional diversity of a plant community [38]. In this study, we controlled the number of life-forms planted in replicated green roof modules, while also measuring green roof services (summer roof cooling and water capture) throughout the third growing season in order to determine the role of plant type and diversity in the functioning and stability of green roofs.

## Methods

### Study Site

We used a roof approximately 5 m above ground level on the Saint Mary's University campus in Halifax, Nova Scotia (44°39′N, 63°35′W). Halifax has a cold, humid maritime climate (Table 1) and during the study period (May-August 2009) average monthly air temperature ranged from 10.2°C–19.4°C and total rainfall reached 532.4 mm [39]. The site received shade for portions of the day due to buildings 1–3 stories taller adjacent to the roof along the west, south and east sides. Prior to this study, the roof structure consisted of a layer of grass growing in approximately 40 cm of clay soil, over a waterproofing membrane that covers a concrete slab. Because there was already a layer of turfgrass on the roof, grey weed barrier fabric (Quest Plastics Ltd., Mississauga, ON, Canada) was laid over the grass (under our green roof modules) to minimize any influence the grass might have potentially had on the measured variables.

**Table 1:** Thirty-year historic climatic averages from regions where the effects of plant selection on green roof performance has been researched (except heating and cooling degree days which are averages from 2007-2009)[55]-[60].

		Daily Temp (°C)			Heating Degree Days^a^	Cooling Degree Days^b^	Rainfall (mm)	Sunlight (hrs)	Relative humidity (%)	
City	Month	Min	Max	Mean	Monthly Sum	Monthly Sum	Monthly Sum	Daily Avg	AM	PM
**Halifax, CAN**	**May**	5.5	14.1	9.8	196.0	7.3	118.1	6	76.0	62.0
	**June**	10.5	19.4	15.0	59.3	21.7	108	7	77.0	63.0
	**July**	14.2	22.9	18.6	17.3	69.0	105.9	8	81.0	64.0
	**August**	14.8	23.0	18.9	15.7	63.7	98.3	7	82.0	65.0
	**September**	11.4	19.0	15.2	73.7	17.0	107.1	6	82.0	65.0
	**Avg.**	11.3	19.9	15.5	–	–	–	7.0	79.0	63.5
	**Total**	–	–	–	362.0	178.7	537.4	–	–	–
	**Source**	1	1	1	5	5	1	3	3	3
**Toronto, CAN ** [**52**]	**May**	9.9	18.5	14.2	125.7	9.3	73.3	7	73.0	55.0
	**June**	14.8	23.5	19.2	23.3	46.0	71.5	9	78.0	58.0
	**July**	17.9	26.4	22.2	8.3	68.7	67.5	9	79.0	56.0
	**August**	17.3	25.3	21.3	2.3	99.3	79.6	8	83.0	58.0
	**September**	13.2	20.7	17	20.0	38.7	83.4	7	87.0	60.0
	**Avg.**	14.6	22.9	18.8	–	–	–	8	80	57.4
	**Total**	–	–	–	179.6	262.0	375.3	–	–	–
	**Source**	1	1	1	5	5	1	3	3	3
**Vancouver, CAN ** [**53**]	**May**	9.5	16.8	13.2	107.7	6.3	86.7	8	88.0	63.0
	**June**	12.2	19.6	15.9	46.7	16.7	69.9	7	87.0	65.0
	**July**	14.1	22.0	18.1	11.7	52.0	49.1	9	89.0	62.0
	**August**	14.4	22.3	18.3	14.7	34.7	48.3	8	90.0	62.0
	**September**	11.6	19	15.4	57.3	9.0	71	6	92.0	72.0
	**Avg.**	12.4	19.9	16.2	–	–	–	7.6	89.2	64.8
	**Total**	–	–	–	238.1	118.7	325	–	–	–
	**Source**	1	1	1	5	5	1	3	3	3
**Portland, USA** [**54**]	**May**	8.6	19.3	13.9	75.3	40.3	60.5	8	70.0	58.0
	**June**	11.4	22.6	17.1	31.3	54.3	40.4	10	73.0	60.0
	**July**	13.8	26.3	20.1	8.7	141.7	18.3	10	75.0	63.0
	**August**	14.1	26.5	20.3	9.3	113.0	23.6	9	78.0	62.0
	**September**	11.4	23.7	17.5	33.0	70.0	41.9	8	79.0	63.0
	**Avg.**	11.9	23.7	17.8	–	–	–	9	75	61.2
	**Total**	–	–	–	157.6	419.3	184.7	–	–	–
	**Source**	2	2	2	5	5	2	3	3	3
**Lansing, USA ** [**27**]	**May**	7.1	20.8	13.9	100.3	33.3	68.8	8	73.0	59.0
	**June**	12.4	25.6	19.0	21.7	100.7	91.4	10	74.0	58.0
	**July**	14.7	27.8	21.3	15.0	118.7	68.1	9	73.0	53.0
	**August**	13.9	26.5	20.2	15.0	122.0	87.9	9	77.0	53.0
	**September**	9.4	22.2	15.8	42.7	60.7	88.4	8	80.0	59.0
	**Avg.**	11.5	24.6	18.0	–	–	–	8.8	75.4	56.4
	**Total**	–	–	–	94.7	435.4	404.6	–	–	–
	**Source**	2	2	2	5	5	2	3	3	3
**Sheffield, UK ** [**31**]	**May**	6.7	15.8	11.3	157.7	2.3	61.9	6	71.0	–
	**June**	9.6	18.3	14.0	90.3	6.3	62.6	6	71.0	–
	**July**	11.9	20.9	16.4	60.0	9.0	52.8	6	74.0	–
	**August**	11.7	20.4	16.1	51.0	7.3	66.7	5	77.0	–
	**September**	9.6	17.1	13.3	101.0	1.3	63.2	4	80.0	–
	**Avg.**	9.9	18.5	14.2	–	–	–	5.4	74.6	–
	**Total**	–	–	–	460.0	26.2	307.2	–	–	–
	**Source**	4	4	4	5	5	4	3	3	–
**Berlin, GER ** [**41**]	**May**	8.2	18.6	13.5	77.7	28.7	52.5	8	80.0	57.0
	**June**	11.4	21.8	16.7	34.3	51.3	65.5	8	80.0	58.0
	**July**	12.9	23.1	17.9	18.7	73.7	46.0	8	84.0	61.0
	**August**	12.4	22.8	17.2	20.7	71.0	55.6	7	88.0	61.0
	**September**	9.4	18.7	13.5	75.7	16.7	49.7	6	92.0	65.0
	**Avg.**	10.9	21.0	15.8	–	–	–	7.4	84.8	60.4
	**Total**	–	–	–	227.1	241.4	269.3	–	–	–
	**Source**	6	6	6	5	5	6	3	3	3

a Heating degree days calculated as the number of days or fraction thereof for which temperatures were below 15°C times (15 (°C) – temperature).

b Cooling degree days calculated as the number of days or fraction thereof for which temperatures were above 18°C times (temperature (°C) - 18).

### Green Roof System

We used a modular green roof system, which consists of self-contained units that can be assembled and planted *ex situ* and later installed on the roof [20]. Each module represented a single replicate, and all modules had the same growing medium and protective layers, only the planted vegetation differed between modules (described below). We used 150 Botanicals Nursery LLC (Wayland, MA, USA) modules (Fig. S1), each consisting of a square, plastic, free-draining tray (36 cm ×36 cm ×12 cm) lined with a composite non-woven water-retention layer (Huesker Inc., Charlotte, NC, USA), followed by an Enkamat (Colbond Inc., Enka, NC, USA) above to act as a drainage/filter layer which was topped with growing medium. We used a commercially available green roof growing medium (Sopraflor X, Soprema Inc., Drummondville, QC, Canada) to a depth of ∼6 cm (above the Enkamat). The growing medium consisted of crushed brick, blond peat, perlite, sand and vegetable compost, had a pH of 6.0–7.0, a total porosity of 60–70%, a bulk density of 1150–1250 kg·m^−3^ and an organic matter content (by dry weight) of 5–10% (Soprema Inc., Drummondville, QC, Canada).

### Plant Material

We planted 15 species (Table 2): 11 native to Nova Scotia; three non-natives (*Poa compressa*, *Sedum acre* and *Sedum spurium*) commonly used on green roofs in Europe and North America [40], [41], and one non-native (*Spergularia rubra*), chosen based on its growth form. While we initially thought that all species represented perennials, we found that two creeping forbs *Minuartia groenlandica* and *S. rubra* were functionally annual.

**Table 2:** Summary of community and ecosystem properties in green roof modules planted with no plants, monocultures or one, three or five life-form groups.

Planted treatment	n	Planted species richness	Realized species richness	Canopy diversity (H′)	Aboveground Biomass index	Aboveground biomass variability	Surface temperature (°C)	Water capture (kg)	Water loss (kg)	Multi-functionality index	Proportion Radiation reflected
no plants	9	0	na	na	na	na	26.59±0.39	**0.86±0.03**	**0.92±0.02**	−0.63±0.52	0.16±0.0016
*Campanula rotundifolia* (tall forb)	3	1	1.0±0.00	0.00±0.00	73.94±3.29	0.37±0.063	25.86±0.19	0.80±0.02	0.86±0.04	−1.09±0.19	0.18±0.0029
*Danthonia spicata* (grass)	3	1	1.0±0.00	0.00±0.00	94.06±10.31	0.31±0.012	24.56±0.40	0.73±0.01	0.78±0.01	−1.57±0.34	0.18±0.0022
*Deschampsia flexuosa* (grass)	3	1	1.0±0.00	0.00±0.00	**169.17±24.75** 1	**0.25±0.030**	23.82±0.87	0.68±0.03	0.74±0.05	−1.97±0.36	0.18±0.0036
*Empetrum nigrum* (creeping shrub)	3	1	1.0±0.00	0.00±0.00	46.11±8.42	0.30±0.045	25.92±0.51	0.79±0.05	0.84±0.09	−1.44±0.87	0.17±0.0015
*Gaultheria procumbens* (creeping shrub)	3	1	1.0±0.00	0.00±0.00	9.28±2.19	0.51±0.030	26.07±1.00	0.76±0.02	0.83±0.05	−1.95±0.75	0.16±0.0007
*Poa compressa* (grass)	3	1	1.0±0.00	0.00±0.00	**118.72±25.28**	0.48±0.132	23.50±0.35	0.81±0.01	0.86±0.05	0.42±0.12	**0.19±0.0062**
*Plantago maritima* (tall forb)	3	1	1.0±0.00	0.00±0.00	52.39±6.79	0.39±0.024	25.48±0.26	0.82±0.01	0.89±0.02	−0.66±0.35	0.17±0.0033
*Sedum acre* (succulent)	3	1	1.0±0.00	0.00±0.00	75.56±15.55	**0.25±0.042**	**23.36±0.58**	0.73±0.04	0.77±0.08	−0.87±0.90	0.18±0.0036
*Solidago bicolor* (tall forb)	3	1	1.0±0.00	0.00±0.00	74.22±3.65	**0.21±0.027**	**21.77±0.49**	**0.82±0.02**	**0.94±0.04**	**1.45±0.08**	**0.21±0.0027**
*Sagina procumbens* (creeping forb)	3	1	1.0±0.00	0.00±0.00	93.17±27.86	0.30±0.038	24.93±0.54	**0.83±0.03**	**0.93±0.06**	−0.17±0.67	0.18±0.0071
*Rhodiola rosea* (succulent)	3	1	1.0±0.00	0.00±0.00	8.72±2.56	0.55±0.234	25.47±0.49	0.70±0.00	0.77±0.02	−2.51±0.28	0.16±0.0015
*Sedum spurium* (succulent)	2	1	1.0±0.00	0.00±0.00	29.75±10.08	0.55±0.063	25.37±1.17	0.78±0.03	0.87±0.02	−1.24±0.26	0.18±0.0074
*Vaccinium vitis-idaea* (creeping shrub)	3	1	1.0±0.00	0.00±0.00	0.33±0.33	na	26.84±0.50	0.77±0.01	0.84±0.04	−2.16±0.18	0.16±0.0005
Creeping shrubs	5	3	3.0±0.00	0.33±0.13	18.30±1.27	0.40±0.061	26.05±0.29	0.80±0.02	0.87±0.02	−1.31±0.23	0.16±0.0007
Creeping forbs	5	3	1.0±0.00	0.00±0.00	44.27±9.75	0.33±0.064	26.17±0.39	0.76±0.00	0.84±0.03	−1.95±0.22	0.17±0.0028
Grasses	5	3	3.0±0.00	0.55±0.11	**112.90±11.09**	0.35±0.053	23.68±0.49	**0.90±0.05**	0.88±0.04	**1.67±0.64**	**0.19±0.0041**
Succulents	5	3	2.6±0.24	0.41±0.11	57.47±3.50	**0.19±0.026**	23.45±0.70	0.76±0.04	0.80±0.05	−0.42±0.42	0.18±0.0040
Tall forbs	5	3	3.0±0.00	0.50±0.12	63.27±5.98	0.32±0.040	**22.71±0.81**	**0.82±0.03**	**0.94±0.04**	**0.97±0.74**	**0.20±0.0040**
Creeping shrubs, creeping forbs, succulents	5	9	6.0±0.45	0.97±0.07	58.03±3.12	**0.22±0.016**	23.56±0.38	0.81±0.03	0.85±0.04	0.34±0.44	0.18±0.0023
Creeping shrubs, creeping forbs, tall forbs	5	9	6.2±0.20	0.80±0.18	65.60±4.61	0.37±0.045	**23.29±0.37**	**0.85±0.01**	0.90±0.06	**1.03±0.35**	**0.19±0.0045**
Creeping shrubs, grasses, creeping forbs	5	9	5.0±0.32	0.49±0.15	**120.33±8.72**	0.42±0.073	23.55±0.33	**0.83±0.02**	**0.93±0.03**	**0.67±0.40**	0.19±0.0030
Creeping shrubs, grasses, succulents	5	9	6.8±0.37	1.15±0.11	**120.57±8.80**	**0.26±0.031**	23.52±0.46	0.79±0.02	0.90±0.02	−0.02±0.41	**0.20±0.0025**
Creeping shrubs, grasses, tall forbs	5	9	7.4±0.60	1.11±0.10	**107.97±13.05**	0.31±0.038	**22.77±0.86**	0.80±0.01	**0.95±0.04**	**0.63±0.54**	**0.20±0.0037**
Creeping shrubs, tall forbs, succulents	5	9	6.8±0.58	1.12±0.08	73.67±4.58	**0.25±0.045**	**22.09±0.30**	**0.82±0.03**	**0.96±0.05**	**1.40±0.47**	**0.20±0.0037**
Creeping forbs, tall forbs, succulents	5	9	6.0±0.45	1.31±0.08	57.53±4.54	**0.21±0.022**	**22.80±0.62**	0.80±0.01	0.89±0.02	**0.60±0.41**	0.19±0.0031
Grasses, creeping forbs, succulents	5	9	5.8±0.37	1.24±0.06	**106.00±8.51**	0.34±0.042	**23.12**±**0.20**	0.81±0.03	0.86±0.03	0.56±0.44	0.19±0.0034
Grasses, creeping forbs, tall forbs	5	9	5.2±0.49	1.20±0.10	**100.67±2.90**	0.31±0.058	**23.16±0.28**	0.80±0.02	**0.93±0.04**	0.40±0.42	**0.20±0.0030**
Grasses, tall forbs, succulents	5	9	5.8±0.37	1.30±0.09	**108.30±10.04**	**0.23±0.047**	**22.71±0.34**	**0.83±0.01**	**1.01±0.04**	**1.20±0.18**	**0.20±0.0023**
Grasses, creeping forbs, tall forbs, succulents, creeping shrubs	20	15	8.7±0.37	1.37±0.04	**101.14±3.14**	**0.25±0.019**	**22.99±0.27**	**0.82±0.01**	**0.93±0.02**	**0.84±0.25**	0.19±0.0019

1 The best ten treatments for each performance measure are in bold (for aboveground biomass variability and surface temperature, optimal functioning is indicated by the lowest values).

Native species were selected from Nova Scotian rocky coastal barren habitats, with the assumption that these species would be best suited to the extensive green roof environment: shallow soil, high winds, intermittent flooding and drought, and absence of tree cover [42], based on their natural growing conditions. Species selected had all been propagated *ex situ* previously by our research group and we only used species that had relatively high germination and growth rates in greenhouse conditions [32]. The native plants selected thus represent a non-random selection of all species occurring in local coastal barrens habitat, mainly chosen based on horticultural criteria and life-form.

The 15 species consisted of three species from each of five life-form groups chosen to examine the influence of life-form diversity in green roof performance: creeping shrubs, creeping forbs, grasses, succulents, and tall forbs (Table 2). Seeds and cuttings were propagated in a greenhouse between 2006 and 2007. Due to a shortage of seedlings, some individual plants of *Gaultheria procumbens*, *Vaccinium vitis-idaea* and *P. compressa* were collected from coastal barrens within 40 km of Halifax in May 2007. Collected plants were put into plug trays using Pro-Mix potting soil (Premier Horticulture, Riviere-du-Loup, QB, Canada) and allowed to establish for at least two weeks prior to planting. Plant size differed between and within species at the time of planting. To control for differences within species, we planted a mix of both relatively large and small plants in all treatments including that particular species. All modules were planted between June 5–19 2007 and watered three to six times per week until July 18 2007. Thereafter they received water primarily through rain events, only receiving 750 mL of supplemental irrigation each on three separate occasions (July 26, July 27 and August 3 2007). In summer 2008, as part of a water balance study, each module received 650 mL of supplemental water on five occasions and 1300 mL on three occasions. Plants that had died after planting (primarily individuals of *Empetrum nigrum*, *Campanula rotundifolia* or *V. vitis-idaea*) were replaced between June 20–29, 2007 (first growing season), after which, individual deaths were simply recorded. During the three growing seasons, individuals of species not planted in a particular module were removed on a regular basis.

### Experimental Design

In order to compare treatments differing in species composition and life-form diversity, we used a randomized complete block design. We planted three replicates of each species in monoculture (in blocks 1, 3 and 5), five replicates (1 per block) of each of the one life-form group plantings and of all the possible combinations of three life-form groups (10 combinations), and finally, 20 replicates (4 per block) of the combination of all five groups (Table 2). When one, three or all five life-form groups were included in replicate modules, all three species within that life-form group were planted. Additionally, ten unvegetated modules with growing medium (two per block) served as controls.

We planted 21 individual plants in each module (regardless of the number of life-form groups present) in four rows of four plants (on 9 cm centers) and a center row of five plants (on 7 cm centers). For unvegetated modules, 21 potting soil plugs were inserted into the growing medium in the same pattern. Twenty-one plants per module was chosen as the density level because it represented a reasonable (in terms of growing space) multiple of the number of species per life-form group. The planting sequence involved alternating life-form groups (if more than one group was included in a module), with the life-form and species pattern being randomly chosen (without replacement) until all species to be included had been selected once, after which, the same pattern was repeated throughout the module until a total of 21 plants had been included.

We arranged the modules in five long, narrow blocks, each block being two modules wide. We placed the modules such that they were in contact with as many other modules as possible (up to eight) to reduce edge effects, but we also allowed for walkways between blocks for access. We oriented the blocks approximately north to south since the primary sunlight and shadow gradient (from surrounding buildings) occurred along a west to east orientation across the site (Fig. S2). We randomly ordered the modules within blocks and rotated them within a block at least six times during each of the three growing seasons.

### Measurement of Green Roof Ecosystem Services and Properties

During the third growing season (May-August 2009) we quantified ecosystem properties/processes in each module. We used Taylor 9878 Slim-Line Pocket Digital Thermometers (Commercial Solutions Inc., Edmonton, AB, Canada) to measure the growing medium surface temperatures (probe inserted ∼1 cm below the surface), near the center of each module. We took these readings on three clear sunny days: May 27 (air temperature ∼15°C), July 31 (air temperature ∼27°C) and August 27 (air temperature ∼21°C), as close to solar noon as possible when modules were sunlit (between 11:00 am and 1:00 pm AST). Heat gain in buildings via surface effects is largely a function of the magnitude of heat flux [flux density of sensible heat, (q_E_) (W/m^2^)]. Heat flux into the building from the external environment is a function of the temperature differential (degrees K) between the interior and exterior environment and the thermal conductivity of the medium through which the energy is passing, *U* (Wm^−1^ K^−1^) [28], [33] which can be coarsely approximated: 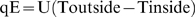



Due to the difficulty in replicating heat flux measurements in our modular system, we used an adjacent experimental built-in green roof (equipped with thermocouples and heat flux transducers) on the same building with the same growing medium and similar plant communities to quantify heat flux, and relate it to measured surface temperatures in the modular system (details in Supporting Information [Text S1, Fig. S3]).

To estimate albedo (reflectivity), we measured incident and reflected solar radiation for each module on four days under clear sky conditions (May 26, July 10, August 14, August 28 2009) and as close as possible to solar noon (between 11:00 am and 1:00 pm AST). We removed each module from the experimental array (at least 2 m away) and placed it on top of grey colored weed barrier fabric (Quest Plastics Ltd., Mississauga, ON, Canada) to ensure that the grass on the study roof and adjacent modules did not contribute to the measured reflectance values. We measured incoming and reflected solar radiation with a LI-200SL pyranometer (LI-COR Biosciences, Lincoln, NE, USA), for which the spectral response includes the 400–1100 nm range. The pyranometer was mounted on a stand 50 cm from the ground (approximately 35 cm from the surface of the growing medium in the modules) and was rotated 180° in order to measure both incoming and reflected radiation. Here, albedo is expressed as: reflected radiation/incoming radiation. The same method was applied to the built-in roof panels on June 1 and August 20 2009 [Text S1].

To quantify water capture, we added 1300 mL of tap water (equivalent to a 10 mm rain event) to each module four times (June 1, July 8, August 4 and August 26 2009). Water was added to the surface of the growing medium in each module over a period of approximately 30 s. We weighed each module prior to adding water and again ten minutes post-watering. The ten-minute delay ensured that any excess water (runoff) drained out of the module and that the growing medium was near field capacity. Capture was expressed as the pre-watering weight subtracted from the post-watering weight (kg of water captured). Our estimate of water capture is likely conservative since water was applied rapidly (simulating a high intensity rain event that would likely occur over a longer time period if the same amount of water fell during a natural event). Furthermore, rain can be intercepted by the vegetation canopy and re-evaporated without entering the growing medium. We did not quantify this potential capture in our experiment as water was added at the base of the vegetation.

We quantified the amount of water lost to evapotranspiration by re-weighing each module 48 hours after the water additions described above. Water loss was calculated as the final weight 48 hours after addition subtracted from the post-watering weight (kg of water lost). There were no natural rain events in the 48 hour periods, so any change in weight can be attributed to the sum of evaporation from the growing medium and transpiration from plant tissues. This method does not allow us to describe the total amount of evapotranspiration over the summer but does provide an index of water loss rates during relatively wet conditions (which are common in the Canadian Maritimes). Thus, our estimates underestimate the effect of transpiration and the difference between unvegetated controls and vegetated modules since the vegetated modules should have been able to reduce soil water content below that of unvegetated controls to a greater extent once the substrate surface became dry [32].

We used a 3-dimensional pin-frame (Domenico Ranalli, Regina, SK, Canada) with 16 pins (each with a diameter of 6 mm, arranged 5 cm apart in a square array) to derive an index of aboveground biomass in each module on May 26, June 10, June 25, July 7, July 17 and August 5 2009. The total number of plant contacts with each pin, regardless of species, was summed across all rods for each module to generate an aboveground biomass index for each sampling day. A more detailed assessment was made twice during the growing season, in May and again in August, where the total number of plant contacts *per species* on each of the 16 pins was summed within each module.

### Statistical Analyses

Two modules (one growing-medium only control and one monoculture of *S. spurium*) were destroyed due to vandalism during the first growing season and were not included in analyses. The monoculture treatments of the two creeping forbs that were functionally annual showed almost no seedling recruitment in the second and third growing seasons and no observable plant cover, therefore, these treatments were also removed from analyses. For each module we calculated the average value of each ecosystem property over all times sampled (temporal mean). The temporal variability of aboveground biomass was expressed as the coefficient of variation using the same values we used to calculate the temporal mean. In order to determine whether the replicates that optimized water capture or temperature reductions scored well in both functions, we created an index of ecosystem multifunctionality [43]. We first took the negative of surface temperature (-1 x surface temperature) such that modules optimizing this ecosystem service had high values, then standardized and centred both the negative of surface temperature and untransformed water capture to create two variables of mean zero and unit variance, then summed the two transformed variables. Replicate modules with high water capture and low surface temperatures (optimizing both services) have high values of this index. This index weights each of the two component functions equally, which may not accurately portray their relative economic or environmental importance [44], but at the least, this index provides a general indication of which treatments are best able to provide multiple benefits.

The ecosystem functions in vegetated modules were initially compared among treatments using general linear models, with block as a random factor. Since the block effect was only significant for water loss, we used linear regression to relate planted species richness to each function. To describe the relationships between ecosystem properties, some of which represent primary green roof services (surface temperature and water capture) and others being secondary functions, we used multiple linear regression, with both forward and backward selection (stepAIC function in R) (Table S1). Besides the measured functions described above, the actual number of species in each module at the beginning of the third growing season (realized richness) and canopy diversity (Shannon diversity: H′, using the total number of contacts within a module as an estimate of species abundance), calculated as the average of the two detailed aboveground biomass index values for each module (described above), were also used as potential predictors in these regressions (Table S1). We used the standardized regression coefficients from the multiple regression analyses to construct a path diagram linking various ecosystem properties and processes.

## Results and Discussion

**Figure 1:**
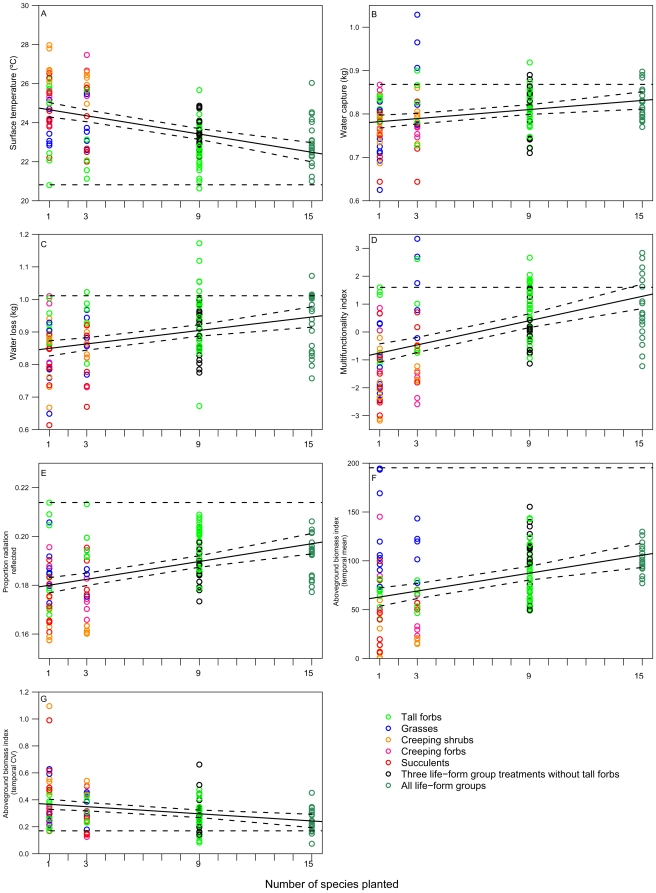
The dependence of ecosystem services and properties in individual roof modules on planted species richness. The solid line is fitted from a regression (±95% CI) of the ecosystem property on the number of planted species (unplanted controls omitted). This is the same data as in Figure 1, without the substrate-only controls, and with number of species planted, instead of the number of functional groups on the x-axis to permit comparison in a regression framework. (A) R^2^_adj_ = 0.27, F_1,131_ = 54.63, P = 1.20×10^-11^; (B) R^2^_ adj_ = 0.07, F_1,131_ = 11.58, P = 8.80×10^-4^; (C) R^2^_ adj_ = 0.12, F_1,131_ = 18.47, P = 3.33×10^-5^; (D) R^2^_ adj_ = 0.23, F_1,131_ = 41.43, P = 2.11×10^-9^; (E) R^2^_ adj_ = 0.19, F_1,131_ = 31.97, P = 9.36×10^-8^; (F) R^2^_ adj_ = 0.14, F_1,131_ = 22.45, P = 5.54×10^-6^; (G) R^2^_ adj_ = 0.08, F_1,129_ = 11.75, P = 8.18×10^-4^. The horizontal dashed line represents the value of the best performing monoculture replicate. For (A) surface temperature, and (G) temporal variability in aboveground biomass, lower values indicate improved performance. In treatments with three life-form groups, those modules containing the tall forb group are in green, whereas combinations not including tall forbs are in black. Monoculture treatments are grouped by life-form such that all three species within a life-form group have the same colour.

Roof surface temperature was greatest in conventional roof controls (mean ± SE: 38.03±0.75°C); the growing-medium-only roof modules reduced temperature by over 10°C, with monocultures and one life-form groups providing an additional 2°C reduction, on average (Table 2)(Fig. 1A). The three and five life-form group treatments outperformed the lower diversity treatments by an additional ∼1.5°C, on average (Fig. 1A), but the best monocultures showed equivalent performance to the best mixtures. Of the monocultures, *Sedum acre* (succulent) and *Solidago bicolor* (tall forb) were within the top ten treatments (Table 2), and the combination of all three tall forbs was the only single life-form group in the top ten. The rest of the best performing treatments were three life-form mixtures, mostly containing tall forbs, and mixtures with all five life-forms. If roof cooling were the major impetus for green roof construction in our region, we might recommend planting only *S. bicolor*, as it had the best temperature performance of all treatments (Table 2).

A reduction in roof surface temperature of 1.5°C corresponds to a reduction in heat flux into the building of 7.14±0.38 W/m^2^ in our system [Text S1]. For perspective, in a study where heat flux through a control roof was 30 W/m^2^ greater than through a green roof, this resulted in a reduction of 75–90% in daily energy consumption for air conditioning [45]. In our system, the corresponding reduction attributable to our best monocultures and mixtures would be up to 20% for a similar building, but our use of different growing media, plant species and control roof composition make further modeling necessary to quantify the energy savings attributable to our treatments.

**Figure 2:**
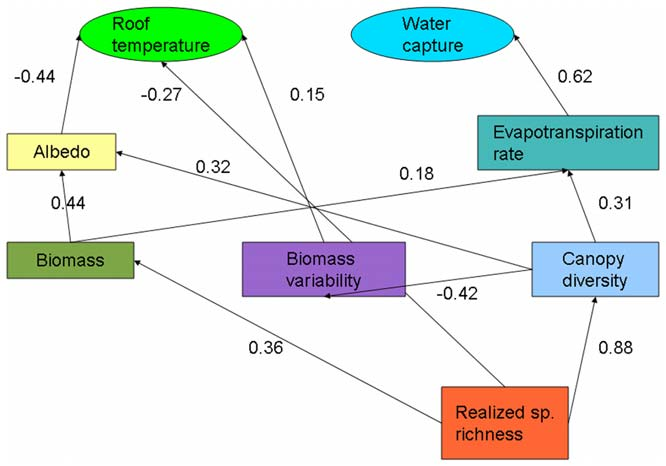
Path diagram showing correlations between ecosystem and vegetation properties in a modular green roof system. Standardized regression coefficients are shown for paths indicating a significant correlation at P<0.05 [Table S1].

Surface temperature was mainly correlated with albedo, and independently correlated with species richness and biomass variability, such that modules with greater albedo and richness, and lower biomass variability had the lowest temperatures (Fig. 2)(Table S1). Albedo itself was related to biomass and diversity where greater biomass corresponded to overall greater reflection of solar radiation. The independent effects of canopy diversity on albedo (Fig. 2) require further investigation to determine the mechanisms operating. Since the indicator of the roof cooling service in this study was the temporal mean of surface temperatures taken during sunny conditions, it is likely that modules with more consistent canopy biomass had lower average temperatures, because modules with more variability in biomass had higher temperatures when biomass was low.

Other studies have emphasized evapotranspiration [25], [30] and increased insulation value [24] as the main functions of green roofs in reducing heat flux through the roof, but more recent green roof thermal models also incorporate the greater albedo (reflectivity) of vegetation compared to conventional roof surfaces [28]. Surprisingly, evapotranspiration (here measured as total water loss), was not correlated with surface temperature (Fig. 2), but this may simply result from our measurements of water loss which only spanned the first 48 hours after water addition events (thus underestimating total water loss during drier conditions, and only highlighting species and mixtures that had high evapotranspiration rates under relatively wet conditions). Here, the conventional roof had an albedo of 0.066±0.006, compared with ∼0.158 for growing medium-only controls (Table 1) and 0.180–0.195 for vegetated modules (Fig. 1E). Our study provides empirical evidence that reflectivity can be an important determinant of green roof cooling.

Recent modeling studies suggest that significant reductions in global average temperatures can be obtained by increasing the albedo of crop plant leaf surfaces by 0.04, via selecting reflective varieties [35]. Here we show that albedo can be increased by more than 0.03 over the worst performing monocultures, by selecting the best monocultures (*Poa compressa* or *Solidago bicolor* (Table 2), or by planting three or five plant life-form groups, instead of low diversity vegetation.

The three and five life-form group treatments captured more water than the monoculture treatments on average (Fig. 1B) but not more than growing-medium only controls (Table 2), with the combination of three grasses, some of the three life-form groups, and the combination of all five showing the best performance (Table 2). Water lost due to evaporation and transpiration followed a similar pattern (Fig. 1C). The best-performing high diversity modules in temperature reduction and water capture functions simultaneously optimized both functions (Fig. 1D). Whereas the tall forb life-form group showed high performance in temperature reduction, water capture, evapotranspiration and albedo functions (Table 2), the grasses had high biomass production, and the succulents had high temporal constancy of biomass. No single life-form group optimized all functions equally, but combining tall forbs, grasses and succulents resulted in performance in the top ten for all functions (Table 2). Multifunctionality is a relatively unexplored benefit of diverse ecosystems, and while we only tested two distinct services in this ecosystem, these results should encourage further exploration of the potential for high plant diversity green roofs to provide a greater range of benefits than less diverse plantings [43].

Other studies have also found a minimal effect of vegetation on water capture over and above that of the growing medium [27] and our results suggest that low-diversity canopies prevent evaporation, reducing the amount of water that can be captured in subsequent rain events (Fig. 2)(Table S1), presumably by shading the surface of the growing medium [32]. Therefore, two of the primary green roof functions are linked: transpiration both cools the roof surface and removes water from the growing medium, allowing for greater stormwater capture.

Even though greater aboveground biomass in high diversity treatments likely also reduces evaporation from the growing medium, overall water loss due to transpiration appears to cancel out this effect. Both biomass and canopy diversity were independently correlated with evapotranspiration (water loss), with greater total area for gas exchange likely made possible by greater canopy biomass. The direct effect of canopy diversity on evapotranspiration could be due to temporal complementarity of water uptake [32], but direct measures of stomatal conductance over time are required to examine this possible mechanism.

Canopy reflectivity (albedo), and aboveground biomass were also maximized on average by life-form group mixtures (Figs. 1E, F), but the best monocultures outperformed the best mixtures indicating that there is no overall positive effect of life-form diversity on these functions. Temporal variability of biomass was decreased by planting three or five life-forms (Fig. 1G) (Table 2), but the effects were fairly small. Aboveground biomass was maximized in all treatments containing grasses, with *Deschampsia flexuosa* producing the most aboveground biomass. Temporal stability of aboveground biomass was maximized in *Sedum acre* (succulent) monocultures and in all treatments containing the succulent group.

Two principal mechanisms are responsible for positive biodiversity-ecosystem functioning relationships: the sampling effect, where mixtures perform as well as the best monoculture because they contain the top performing species [36], [46], [47], and transgressive overyielding, where some species-rich replicates outperform the best monoculture, due to niche complementarity or facilitation [36], [48]. Among the ecosystem properties measured here, for surface temperature, reflectivity, and aboveground biomass, the relationships between diversity and function appear to represent sampling effects, where the best replicate monocultures are equivalent to, or exceed the best high diversity treatments (Fig. 1A,E,F). For temperature and albedo, it is clear that treatments containing the tall forb group are the best performers (Fig. 1A,E), and this is likely due to the presence of *S. bicolor*, which grows fast and has relatively large flat leaves, in all of these treatments (the three modules with the lowest temperatures in Fig. 1A are *S. bicolor* monocultures; also see Table 2). On the other hand, Fig. 1 clearly shows that modules containing tall forbs are frequently among the poorest performers for each function as well, thus inclusion of tall forbs was not sufficient to guarantee high performance. Interactions between tall forbs and other life-form groups may promote optimal performance, for example, mixtures containing both tall forbs and grasses seem to perform many functions well (Table 2). The treatments with the greatest biomass, however, were grass monocultures and the single life-form treatment including all three grasses (Fig. 1F), moreover, the three functional group treatments with the greatest biomass all contained grasses. While the sampling effect may be important in determining relationships between biodiversity and ecosystem functioning in this system, different species or groups are responsible for high performance of different functions, thus further supporting the idea that multifunctionality may be an important benefit of plant diversity in green roof systems.

Water capture, water loss, multifunctionality and temporal variation in aboveground biomass show evidence of transgressive overyielding (Fig. 1B,C,D,G). For water capture and the multifunctionality index, the treatments containing all three grasses (single life-form group) performed better than almost all grass monocultures (Fig. 1B,D), thus increasing diversity within a life-form group had a positive effect on functioning in this study (but only in one of the five life-forms tested here). The grass species included have different growth characteristics: *Danthonia spicata* creates dense basal rosettes with low vertical growth, *D. flexuosa* produces a dense bunch of thin leaves at intermediate heights, while *P. compressa* produces long thin tillers which grow tall within the canopy. *D. spicata* and *D. flexuosa* have dense clusters of fibrous roots, whereas *P. compressa* spreads rhizomatously and may have a different pattern of root growth. It is possible that some kind of below-ground spatial complementarity between the three species results in greater potential for water capture, but more work is needed to elucidate the mechanism.

For water loss, and the multifunctionality index, there were several three life-form combinations that outperformed the best monocultures, as well as 25% of the replicates of the five life-form treatment. Potential mechanisms for this overyielding include temporal complementarity of growth phenology or water uptake such that modules with more life-form groups maximized water uptake and possibly temperature performance over a longer period of time [32]. While several modules in the three life-form group treatments appeared to overyield for temporal stability of biomass (Fig. 1G), the particular treatments that performed this function well all contained succulents (Table 2), thus this is also likely a sampling effect. While several of the succulent single life-form group modules appear to outperform their component monocultures (Fig. 1G), this difference is small and further testing is required to determine if there is an advantage of planting more than one succulent species on green roofs.

While we did not vary the number of species separately from the number of life-form groups in this study, the one life-form group treatments can be compared with their component monocultures to evaluate any potential role of species richness within a life-form. In some life-forms, performance in the mixture of all three species is equivalent to the best monoculture in that life-form group, but many others show a dilution effect (Table 2). In such cases, the functionality of the best single species is diluted in the mixtures because they are sharing the plot with poorer performing species, and sufficient time has not elapsed for replacement of the inferior species by the superior [49], [50]. The main diversity effect seen here is thus between, not within life-forms, although further testing with more species within each life form is required to formally examine the effect of within-life-form diversity.

For all of the functions we evaluated, while the five life-form mixtures outperformed some of the three life-form mixtures for some functions (Table 2), planting all five life-forms (15 species) never resulted in a performance advantage compared with the three life-form treatments (9 species) taken as a whole (Fig. 1). This may be the result of a dilution effect where the most diverse treatments have more space taken up by poor performing life-forms, or functional redundancy among life-form groups. Of the three life-form treatments, the best mixture combined succulents, grasses and tall forbs (Table 2), although most of the different combinations of three life-form groups showed statistically equivalent performance. While most extensive green roofs are planted solely with succulents, these results suggest that adding grasses and forbs can improve the green roof services quantified in this study.

Most of the species we used here, including all the grasses and succulents, occur widely across North America and Europe [51], and while their performance may be strongly influenced by local climates, these species and others that are morphologically similar deserve further testing for temperate zone green roofs. Likewise, while each region where green roof research has been conducted has a unique climate, Halifax is comparable in climate to several other areas from where research has been published (Table 1). Specifically, the summer temperatures in Halifax are comparable to Vancouver and Toronto, but Halifax is cooler than Toronto and warmer and wetter than Vancouver. While unique, Halifax does not represent an extreme climate relative to other places where green roof research has emerged, for example, Sheffield, UK is much colder in the summer than Halifax (Table 1). We emphasized summer cooling and stormwater capture in this study, as these benefits have spurred much green roof construction in Europe and North America [20], but future studies will quantify other ecosystem services, such as winter thermal benefits, that may be important in cold temperate climates.

### Conclusions

Our results provide the first evidence that green roof ecosystem services can be improved by increasing the diversity of plant life-forms, however, some life-form combinations did not perform well, thus life-form diversity *per se* is not guaranteed to optimize green roof performance. We can recommend combinations of succulents, grasses and tall forbs for green roof projects as this mixture optimized most of the functions we measured. The differences between the poorest and best performing species, life-forms and mixtures are great enough to suggest that informed plant selection for green roofs should provide significant increases in energy and monetary savings, improvements to urban climates and reductions in greenhouse gas emissions. Further work should characterize the plant traits determining these functions in order to facilitate optimization of green roof performance in different regions, and to clarify the mechanisms underlying the enhanced performance of some of the mixtures described here.

## Supporting Information

Text S1 Methods and Materials. Detailed methods for the built-in green roof system: used here only to derive estimates of conventional roof temperature, heat flux and albedo.
(0.03 MB DOC)

Figure S1 Modular green roof assembly.
(0.09 MB DOC)

Figure S2 Study site for life-form group experiment, showing shadow perpendicular to block arrangement.
(0.87 MB DOC)

Figure S3 Diagram of built-in green roof system with sensor locations.
(0.09 MB DOC)

Table S1 Multiple regression models of ecosystem services and properties for green roof modules planted with monocultures and one, three or five life-form groups (not including growing medium-only controls).
(0.07 MB DOC)
