# Supplementary material for: Plant Species and Functional Group Combinations Affect Green Roof Ecosystem Functions
Source: PLoS One. 2010 Mar 12;5(3):e9677. doi: 10.1371/journal.pone.0009677 (PMC2837352; doi:10.1371/journal.pone.0009677)
Supplement: Text S1 — Methods and Materials. Detailed methods for the built-in green roof system: used here only to derive estimates of conventional roof temperature, heat flux and albedo. (0.03 MB DOC) [file pone.0009677.s001.doc]

Built-in Green Roof System

The built-in green roof equipped to measure heat flux was constructed in spring 2008 on the same roof as the modular experiment. Here, we only use data from this second system to calculate an estimate of heat flux reductions attributable to the vegetation in the modular experiment. The built-in system consisted of four roof panels, each 2.4 m x 2.4 m (8’ x 8’). We placed these extensive roof panels such that the top of the growing medium was flush with the top of the sod that was already on the roof. Extensive green roof drainage containers (ELT EasyGreen, Princeton, ON, Canada) were placed on a plywood base fitted with holes every 3 cm for drainage. Before applying the plywood base, the original soil was removed and backfilled with construction gravel to create a well-draining, level surface. Two of the panels were filled to a depth of 15 cm with the same growing medium used in the modular system described above, and a third panel received 7.5 cm of medium. The fourth panel served as a conventional roof control and had a thin layer of dark grey shingle applied to the plywood base. The green roof panels were all planted (in May and June 2008) with the same mixture of 11 species, which included nine of the 15 species used in the modular experiment (plants were mostly grown in 3” pots, were 4-18 months old, and were planted on 8 cm centers).

During construction of the panels, we embedded thermocouple temperature probes (105T, Campbell Scientific, Edmonton, AB, Canada) in a vertical array at three levels in the conventional roof panel, and five levels in the green roof panels. A heat flux transducer (HFT3, Campbell Scientific, Edmonton, AB, Canada) was placed just under the roof assembly next to the thermocouple at the roof assembly level (under the shingles in the conventional roof and the drainage containers under the green roofs) (Fig. S3). Each panel had the sensor array replicated twice, located approximately 1 m diagonally from the NE and SW corners. Temperature and heat flux data were recorded every 15 minutes using a datalogger (CR 1000, Campbell Scientific, Edmonton, AB, Canada). We calculated average surface temperature (just below the surface of the growing medium for the three green roof panels and below the shingle for the conventional roof panel) and heat flux for each sensor array (two per panel) for the days and times corresponding to those in which modular surface temperatures were measured. We then took the average of the two arrays to obtain a single value for each panel for each day, then averaged these values from the three separate sampling days to generate single values for mean surface temperature and heat flux for each panel (n=4). Finally, we used linear regression to predict heat flux from surface temperature: heat flux (W/m^2^) = 4.76 (surface temperature (ºC)) ± 0.26 – 100.71± 6.95; F_1,2_ = 344.9, R^2^adj. = 0.99, P = 0.0029.

To compare the module growing medium surface temperatures to those of a conventional roof (control), we used the single conventional roof panel described in the previous section and two other conventional roof panels on a raised-bed array on the same roof. The raised-bed array consisted of two conventional roof panels with grey shingles (the same roof membranes as the thermal panels), as well as ten green roof panels (all 4’ x 4’). Each conventional roof panel had a single shielded thermocouple temperature probe (105T, Campbell Scientific, Edmonton, AB, Canada) placed directly on the roof surface.  Panels had been raised 1 m above the roof surface in order to accommodate a flowmeter system (not discussed here). We used temperature data from the two conventional raised bed panels and the conventional roof-level panel described above (n=3) to calculate an average surface temperature over the same time periods in which the modular experiment was sampled.
