# Supplementary figures and images for: Plant Species and Functional Group Combinations Affect Green Roof Ecosystem Functions
Source: PLoS One. 2010 Mar 12;5(3):e9677. doi: 10.1371/journal.pone.0009677 (PMC2837352; doi:10.1371/journal.pone.0009677)

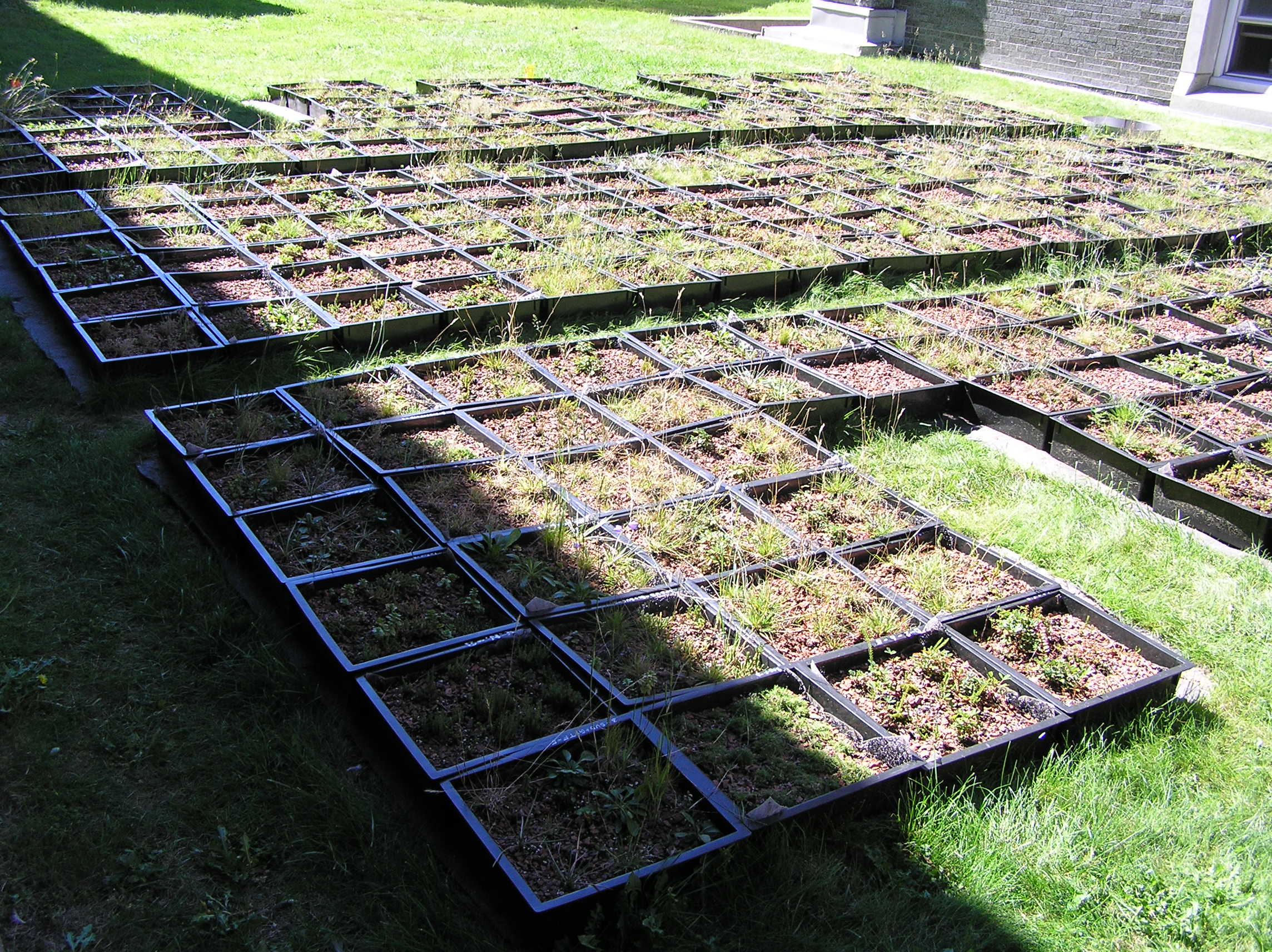

Supplement: Figure S2 — Study site for life-form group experiment, showing shadow perpendicular to block arrangement. (0.87 MB DOC) [file pone.0009677.s003.doc]
