# Supplementary material for: Plant Species and Functional Group Combinations Affect Green Roof Ecosystem Functions
Source: PLoS One. 2010 Mar 12;5(3):e9677. doi: 10.1371/journal.pone.0009677 (PMC2837352; doi:10.1371/journal.pone.0009677)
Supplement: Table S1 — Multiple regression models of ecosystem services and properties for green roof modules planted with monocultures and one, three or five life-form groups (not including growing medium-only controls). (0.07 MB DOC) [file pone.0009677.s005.doc]

**Table S1.**

**Ecosystem property F df Adjusted Variable Coefficient Standardized *P***

**R^2^ Coefficient**

Surface temperature 34.84 3,125 0.44 **Albedo** -53.02 -0.44 <0.0001

**Aboveground biomass**

**variability** 1.56 0.15 0.03

**Realized species richness** -0.15 -0.27 0.0003

(Planted species richness)^1^

(Water loss)

(Aboveground biomass)

(Canopy diversity)

Water capture 77.62 1,127 0.37 **Water loss** 0.38 0.62 <0.0001

(Planted species richness)

(Realized species richness)

(Aboveground biomass)

(Aboveground biomass

variability)

(Canopy diversity)

Water loss 12.32 2,126 0.15 **Aboveground biomass** 4.4 x 10^-4^ 0.18 0.04

**Canopy diversity** 0.05 0.31 0.0005

(Planted species richness)

(Realized species richness)

(Aboveground biomass

variability)

Multifunctionality 35.70 3,125 0.45 **Realized species richness** 0.14 0.28 0.0001

index **Aboveground biomass** 7.9 x 10^-3^ 0.20 0.006

**Water loss** 6.65 0.42 <0.0001

(Planted species richness)

(Aboveground biomass

variability)

(Canopy diversity)

(Albedo)

(Water loss)

Albedo 33.75 3,125 0.43 **Aboveground biomass** 1.5 x 10^-4^ 0.44 <0.0001 **Aboveground biomass**

**variability** -9.2 x 10^-3^ -0.10 0.15

**Canopy diversity** 7.2 x 10^-3^ 0.32 <0.0001

(Planted species richness)

(Realized species richness)

Aboveground 19.47 1,127 0.13 **Planted species richness** 2.84 0.36 <0.0001

biomass (Realized species richness)

Aboveground 17.54 1,127 0.11 **Canopy diversity** -0.09 -0.35 <0.0001

biomass variability (Aboveground biomass)

(Aboveground biomass

variability)

Canopy diversity 171.4 3,125 0.80 **Planted species richness** 0.06 0.50 <0.0001

**Realized species richness** 0.07 0.38 <0.0001

**Aboveground biomass**

**variability** -0.42 -0.11 <0.0001

(Aboveground biomass)

^1^ Variables in brackets are those originally included as potential predictors but not selected in the best model.
