# Supplementary material for: Plant Species and Functional Group Combinations Affect Green Roof Ecosystem Functions
Source: PLoS One. 2010 Mar 12;5(3):e9677. doi: 10.1371/journal.pone.0009677 (PMC2837352; doi:10.1371/journal.pone.0009677)
Supplement: Supplementary file 4 [file pone.0009677.s002.doc]

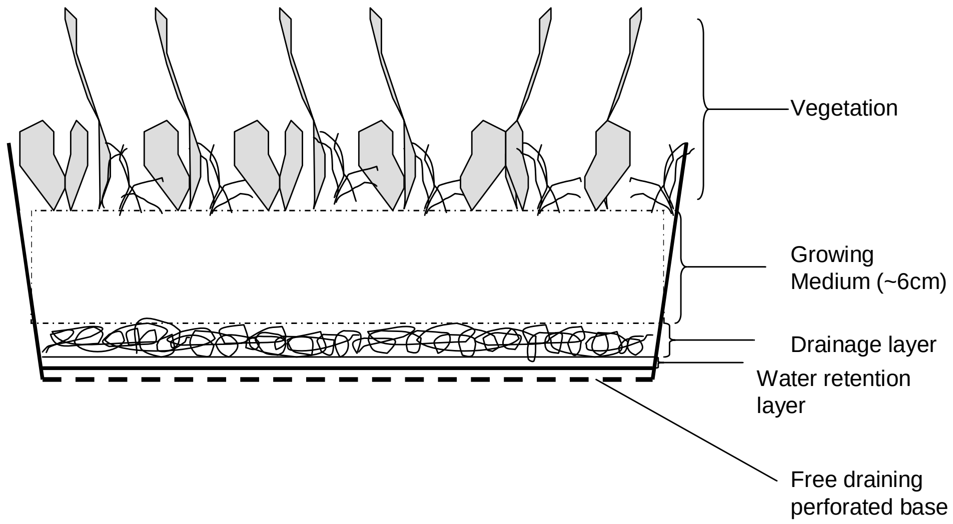
Vegetation
Growing
Medium (~6cm)
Drainage layer
Water retention
layer
Free draining
perforated base
