# Supplementary material for: Plant Species and Functional Group Combinations Affect Green Roof Ecosystem Functions
Source: PLoS One. 2010 Mar 12;5(3):e9677. doi: 10.1371/journal.pone.0009677 (PMC2837352; doi:10.1371/journal.pone.0009677)
Supplement: Supplementary file 5 [file pone.0009677.s004.doc]

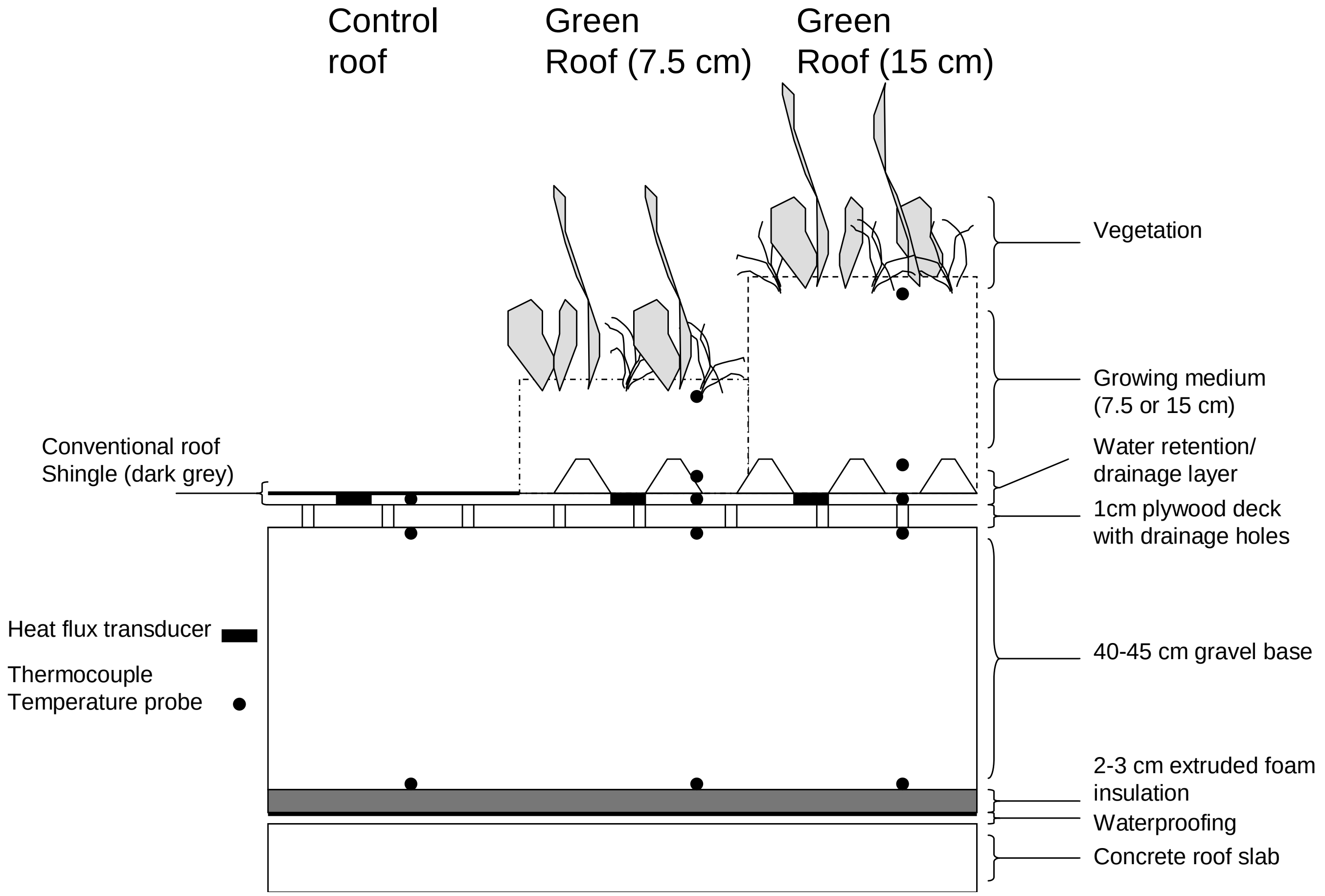
Control
roof
Green
Roof (7.5 cm)
Green
Roof (15 cm)
Vegetation
Growing medium
(7.5 or 15 cm)
Conventional roof
Shingle (dark grey)
Water retention/
drainage layer
1cm plywood deck
with drainage holes
Heat flux transducer
Thermocouple
Temperature probe
40-45 cm gravel base
2-3 cm extruded foam
insulation
Waterproofing
Concrete roof slab
